# Age‐related differences in the relationship between sleep quality and cortical thickness

**DOI:** 10.1002/alz.093110

**Published:** 2025-01-09

**Authors:** Hyun Kim, Yisha Zhang, Benjamin Huber, Elizabeth Rueppel, Terry E. Goldberg, Seonjoo Lee, human connectome project

**Affiliations:** ^1^ Columbia University Irving Medical Center, New York, NY USA; ^2^ New York State Psychiatric Institute, New York, NY USA; ^3^ Columbia University, New York, NY USA; ^4^ Columbia University Medical Center, New York, NY USA; ^5^ Department of Anesthesiology, Columbia University Medical Center, New York, NY USA; ^6^ Mailman School of Public Health, Columbia University, New York, NY USA

## Abstract

**Background:**

While the association between sleep quality and brain health is well established, the role of aging in this relationship is largely unknown. This study aimed to examine the interaction between sleep and age on cortical thickness using samples from large‐scale cohort studies. Age was examined in both linear and non‐linear (quadratic) terms, in order to determine the presence of critical age ranges that may exhibit more significant sleep‐related brain structural changes.

**Method:**

Sample included 700 adults (mean age 59.63 [age range 36‐90], 55.9% female) from the Human Connectome Project‐Aging (HCP‐A). Sleep quality was measured using the Pittsburgh Sleep Quality Index (PSQI). Magnetic resonance imaging (MRI) data was obtained from T1‐weighted structural images using a 3T scanner, and cortical thickness indices were derived using the FreeSurfer pipeline. The interaction between sleep and age on cortical thickness was tested using the moderation analysis using linear regression models, with sex, race, and use of sleep medication as covariates. The same analyses are planned on a separate dataset from the Nathan Klein Institute (NKI) cohort in order to replicate these findings.

**Result:**

There was a significant interaction term between the PSQI and non‐linear age term (age^2^) on the insula (b=0.67, CI=0.03‐1.30, p=.04) and the middle temporal cortical thickness (b=0.58, CI=0.03‐1.13, p=.04), indicating that sleep quality may be associated with cortical thickness distinctly in different age groups. A visual examination of this relationship indicated that cortical thickness for individuals between the ages 53 and 78.

**Conclusion:**

Age may have a significant role in the relationship between sleep and cortical thickness. Middle‐aged and older adults may be most vulnerable to sleep‐related changes in the brain structures. These findings will be tested using a separate dataset.

